# Community-Acquired Methicillin-Resistant *Staphylococcus aureus* Carrying Panton-Valentine Leukocidin Genes: Worldwide Emergence

**DOI:** 10.3201/eid0908.030089

**Published:** 2003-08

**Authors:** François Vandenesch, Timothy Naimi, Mark C. Enright, Gerard Lina, Graeme R. Nimmo, Helen Heffernan, Nadia Liassine, Michèle Bes, Timothy Greenland, Marie-Elisabeth Reverdy, Jerome Etienne

**Affiliations:** * INSERM E0230, Lyon, France; † Centers for Disease Control and Prevention, Atlanta, Georgia, USA; ‡ University of Bath, Bath, United Kingdom; § Princess Alexandra Hospital, Brisbane, Australia; ¶ Antibiotic Reference Laboratory, 12 Wellington, New Zealand; # Laboratoire Bioanalytique-Riotton, Geneva, Switzerland; ** UMR754, Lyon, France

**Keywords:** *Staphylococcus aureus*, leukocidins, enterotoxins, bacterial toxins, community-acquired infections, communicable diseases, emerging bacterial infections, methicillin resistance, research

## Abstract

Infections caused by community-acquired (CA)-methicillin resistant *Staphylococcus aureus* (MRSA) have been reported worldwide. We assessed whether any common genetic markers existed among 117 CA-MRSA isolates from the United States, France, Switzerland, Australia, New Zealand, and Western Samoa by performing polymerase chain reaction for 24 virulence factors and the methicillin-resistance determinant. The genetic background of the strain was analyzed by pulsed-field gel electrophoresis (PFGE) and multi-locus sequence typing (MLST). The CA-MRSA strains shared a type IV SCC*mec* cassette and the Panton-Valentine leukocidin locus, whereas the distribution of the other toxin genes was quite specific to the strains from each continent. PFGE and MLST analysis indicated distinct genetic backgrounds associated with each geographic origin, although predominantly restricted to the *agr*3 background. Within each continent, the genetic background of CA-MRSA strains did not correspond to that of the hospital-acquired MRSA.

Methicillin-resistant *Staphylococcus aureus* (MRSA) are identified as nosocomial pathogens throughout the world (*1*). Established risk factors for MRSA infection include recent hospitalization or surgery, residence in a long-term–care facility, dialysis, and indwelling percutaneous medical devices and catheters. Recently, however, cases of MRSA have been documented in healthy community-dwelling persons without established risk factors for MRSA acquisition. Because they are apparently acquired in the community, these infections are referred to as community-acquired (CA)-MRSA (*2*). CA-MRSA infections have been reported in North America, Europe, Australia, and New Zealand (*3*–*5*). The recent genomic sequence of a CA-MRSA isolate (*6*) indicated the presence not only of a novel smaller variant of the methicillin-resistance locus (SCC*mec* IVa, according to Baba et al. designation [*6*]), but also that of the locus for the Panton-Valentine leukocidin (PVL). The PVL locus is carried on a bacteriophage and is present in only a small percentage of *S. aureus* isolates from France, where this locus is associated with skin infections, and occasionally, severe necrotizing pneumonia (*7*,*8*).

In a recent study, we found that CA-MRSA infections in France are caused by a single clone producing the PVL (*3*). Analysis of a set of CA-MRSA strains from the United States and Australia confirmed the presence of SCC*mec* IVa in most of them, and genetic comparison of the CA-MRSA by multi-locus sequence typing (MLST) indicated that they belonged to five clonal complexes, two of which predominated (*4*). This finding suggested that CA-MRSA have arisen from diverse genetic backgrounds rather than the worldwide spread of a single clone (*4*).

The aim of this study was to determine whether the PVL gene represents a stable marker of the CA-MRSA strains worldwide and whether any other common genetic traits such as toxin gene and the accessory gene regulator (*agr*) profiles can be identified.

## Materials and Methods

### Bacterial Strains

A total of 117 different isolates of CA-MRSA were examined. Community acquisition was defined as growth of the isolates within 48 hours after hospital admission in patients who had no risk factors for nosocomial acquisition, including no hospitalizations or nursing home residence in the year before admission. Thirty-three isolates were from the United States. The U.S. isolates all belonged to the previously identified major CA-MRSA clonal group and included strain MW2 (*2*). They originated from 12 different facilities in Minnesota; two isolates were from North Dakota. Sixty-seven isolates originated from Europe (61 isolates from France and 6 from Switzerland). The French CA-MRSA isolates originated from 10 different hospitals located throughout France, and the Swiss isolates were from the Geneva area. Seventeen isolates were from Oceania and included two different clones: 1) 13 isolates corresponded to the Western Samoan phage pattern strains, also designated the Southwest Pacific clone (*9*) and were isolated from Australia (8 isolates), New Zealand (4 isolates), and Western Samoa (1 isolate) (*5*); 2) 4 other isolates from Australia belonged to a recently described clone designated as the Queensland clone (*10*). The Southwest Pacific clone has two distinct phage-typing patterns known as Western Samoan Phage Pattern (WSPP) 1 and 2 (*5*). The Queensland clone, which cannot be phage typed, was first detected in the southeast Queensland city of Ipswich in 2000 (*10*). Most isolates were from primary skin and soft tissue infections, with some cases of bacteriemia and at least five cases of necrotizing pneumonia (*2*,*3*,*9*,*11*,*12*).

A set of representative hospital-acquired (HA)MRSA isolates was included in the study: 24 from France and 33 from the United States. French HA-MRSA isolates corresponded to those of the major clones isolated throughout France as described by Lelievre et al. (*13*). HA-MRSA isolates were recovered from the same 12 different facilities that CA-MRSA. HA-MRSA from the United States originated from the same geographic area as the CA-MRSA.

### Antimicrobial Susceptibility Testing

The MICs of benzyl-penicillin, oxacillin, gentamicin, tobramycin, kanamycin, chloramphenicol, tetracycline, minocycline, erythromycin, lincomycin, pristinamycin, fusidic acid, rifampicin, ofloxacin, co-trimoxazole, linezolid, mupirocin, vancomycin, and teicoplanin were determined for selected isolates (46 European, 22 U.S., and 13 Oceanian isolates) by using the standardized agar dilution technique as recommended by the French Society for Microbiology (*14*).

### Detection of Accessory Genes by PCR

Using polymerase chain reaction (PCR), we determined the presence of accessory gene regulator (*agr)* allele group (*1*–*4*), SCC*mec* element (I-IV, according to the designation of Oliveira [*15*]), and 22 specific staphylococcal virulence genes (including 16 super-antigenic toxins, 3 hemolysins, and 3 leukocidins), as described previously (*16*).

Amplification of *gyrA* was used as a quality control of each DNA extract and the absence of PCR inhibitors. *S. aureus* strains Fri 913 (*sea*, *see*, *sec*, *tst*, *lukE* *lukD*, *sek*, *sel*, *sep*, and *hlg*), Fri 1151m (*sed*, *sej*, *lukE* *lukD*, *hlgv*, and *hlb*), ATCC 14458 (CCM5757) (*seb*, *lukE* *lukD*, *sek*, and *hlgv*), NCTC 7428 (*sec*, *tst*, *lukM*, *seg*, *sei*, *sem*, *sen*, *seo*, *lukE lukD*, *hlgv*, and *hlb*), A92 0211 (*seg*, *sei*, *sem*, *sen*, *seo*, *eta*, *etb*, *lukE* *lukD*, and *hlgv*), RN6390 (*lukE lukD*, *hlgv*, *hlb*, and *agr1*), RN6607 (*sed*, *seg*, *sei*, *sem*, *sen*, *seo*, *lukE lukD*, *hlgv*, and *agr2*), RN8465 (*seg*, *sei*, *sem*, *sen*, *seo*, *tst*, *hlg*, and *agr3*), RN4850 (*seg*, *sei*, *sem*, *sen*, *seo*, *eta*, *etb*, *lukE lukD*, *hlgv*, and *agr4*), RN 6911(*lukE lukD*, *hlgv*, *hlb*, *agr* null), E-1 (*seg*, *sei*, *sem*, *sen*, *seo*, *lukE lukD*, *eta*, *hlgv, edinB* and *C*), ATCC 49775 (*seg*, *sei*, *sem*, *sen*, *seo*, *lukS* *lukF*, and *hlg*), and ATCC 51811 (FRI 569) (*seh*, *lukE lukD*, *hlb*, and *hlgv*) were used as positive controls for PCR (*17*,*18*). *S. aureus* COL (SCC*mec* I), PER34 (SCC*mec* IA), BK2464 (SCC*mec* II), ANS46 (SCC*mec* III), HU25 (SCC*mec* IIIA), and HDE288 (SCC*mec* IV) were used as controls for characterization of the *mec* element according to Oliveira and de Lencastre (*15*).

The overall genetic background of the isolates was evaluated: 1) by digesting whole cell DNA with *Sma*I macrorestriction enzyme and determining the fragment-size patterns obtained on pulsed-field gel electrophoresis (PFGE) using a contour-clamped homogeneous electric field system on a CHEF DR-II apparatus (Bio-Rad Laboratories, Marnes-la-Coquette, France) as previously described (*19*). Resolved macrorestriction patterns were compared as recommended by Tenover et al. (*20*). Isolates differing by up to three fragments were considered as subtypes of a given clonal type. MLST was performed as described by Enright et al. (*21*). Briefly, seven housekeeping genes were used in the scheme; for each isolate, the alleles at each of the seven loci defined the allelic profile, which corresponded to a sequence type (ST). ST designations were those assigned by the MLST database (available from: URL: http://www.mlst.net).

## Results

### Distribution of Accessory Genes from MRSA in Three Continents

Overall, we detected 12 different virulence genes or gene clusters among the 117 isolates (Table 1). Two gene loci were common to CA-MRSA isolates from all locations. Methicillin resistance was conferred in all 117 CA-MRSA isolates by the truncated SCC*mec* type IV element, and all the isolates contained the PVL locus. In addition, 112 isolates harbored the related *lukE-lukD* genes of another leukocidin frequently recovered from patients with all types of staphylococcal infections (*3*). Most (113 [97%] of 117) isolates were of *agr* type 3.

**Table 1:** Distribution of virulence and resistance determinants in 117 CA-MRSA isolates from three continents^a^

Genes^b^	CA-MRSA isolates from					
	France- Switzerland^c^ n=67 (%)	USA n=29 (%)	USA n=4 (%)	Oceania^d^ Southwest Pacific clone n=13 (%)	Australia Queensland clone n=4 (%)	Total n=117 (%)
Sequence type	80	1	59 or 8	30	93	
PFGE pattern	A1-7	B1-5	D1 & F1	C1-3	E1	
*agr* type	3	3	1	3	3	
SCC*mec* type IVa	67 (100)	29 (100)	4 (100)	13 (100)	4 (100)	117 (100)
Leukocidins PVL genes	67 (100)	29 (100)	4 (100)	13 (100)	4 (100)	117 (100)
*lukE-lukD*	67 (100)	29 (100)	3 (75)	13 (100)	0 (0)	116 (99)
Hemolysins^e^						
*hlg*	0 (0)	0 (0)	0 (0)	13 (100)	0 (0)	13 (11)
*hlg-v*	67 (100)	29 (100)	4 (100)	0 (0)	0 (0)	100 (85)
*hlb*	0 (0)	0 (0)	1 (25)	0 (0)	0 (0)	1 (1)
Enterotoxins *sea*	0 (0)	23 (79)	0 (0)	0 (0)	0 (0)	23 (20)
*seb*	0 (0)	8 (28)	1 (25)	0 (0)	0 (0)	9 (8)
*sec*	0 (0)	20 (69)	0 (0)	0 (0)	0 (0)	20 (17)
*sed-sej*	0 (0)	0 (0)	3 (75)	0 (0)	0 (0)	3 (3)
*she*	0 (0)	29 (100)	0 (0)	0 (0)	0 (0)	29 (25)
*sek*	0 (0)	24 (83)	0 (0)	0 (0)	0 (0)	24 (21)
*egc* ^f^	0 (0)	0 (0)	0 (0)	13 (100)	0 (0)	13 (11)

^a^PFGE, Pulsed-field gel electrophoresis; PVL, Panton-Valentine leukocidin. ^b^Results for toxin genes absent from all community-acquired methicillin-resistant *Staphylococcus aureus* (CA-MRSA) isolates (*see, tst, eta, etb, lukM,* and *edinA*) are not presented. ^c^Isolates from France (61) and Switzerland (*6*). ^d^Isolates from Australia (*8*), New Zealand (*4*) and Western Samoa (*1*). ^e^*hlg*, γhaemolysin gene; *hlg-v*, γ-haemolysin variant gene; *hlb*, β-haemolysin gene. ^f^*egc*: enterotoxin gene cluster, which includes the *seg*, *sei*, *sem*, *sen,* and *seo* gene

The distribution of other genes varied by the continent of origin. The European and Southwest Pacific isolates had consistent, relatively simple, patterns of virulence-associated genes. In addition to the SCC*mec* type IV element, PVL genes, and *lukE-lukD,* all the European isolates were positive for *hlg-v* (the γ-hemolysin variant gene) and the Southwest Pacific isolates were positive for *hlg* (the γ-hemolysin gene) and *egc* (the enterotoxin gene cluster coding for the enterotoxins *seg*, *sei*, *sem*, *sen*, and *seo*) (Table 1). The Queensland isolates had not been tested for the toxin genes, except the PVL locus. In contrast, considerable variability existed among the 33 U.S. isolates. As for the European isolates, the U.S. isolates showed both the presence of *hlg-v* and the absence of *hlg.* However, seven other toxins (the enterotoxins *sea*–*sed*, *sej*, *she,* and *sek*), which were absent in non–U.S. isolates, were variously found in up to 29 (3% to 87%) of these 33 isolates (Table 1).

### Analysis of Genetic Background of CA-MRSA by PFGE

The 117 CA-MRSA isolates clustered into six PFGE clonal types (A to F, Figure). All 67 European isolates grouped into seven related PFGE patterns (subtypes A1-7) distinct from the other isolates. Most of the U.S. isolates belonged to a closely related group of five patterns (subtypes B1-5), although two well-differentiated outliers existed, one (D1) containing three isolates and the other (F1) containing only one isolate, both of which had an *agr* type 1 genotype. The 13 isolates obtained from Australia, New Zealand, and Western Samoa and belonging to the Southwest Pacific clone showed three closely related PFGE patterns (subtypes C1-3) with no geographic association. The four isolates from Australia belonging to the Queensland clone had the same E1 pattern. The phylogenetic tree shown on the left side of the Figure confirms the diversity in PFGE patterns between the CA-MRSA from different continents.

**Figure:**
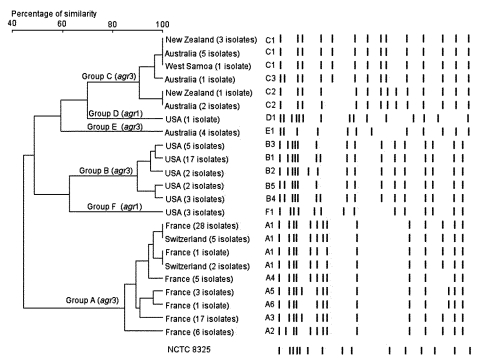
Pulsed-field gel electrophoresis (PFGE) pattern and phylogenetic tree of 117 community-acquired (CA)-methicillin resistant *Staphylococcus aureus* isolates from three continents. *Sma*I macrorestriction patterns were digitized and analyzed by using Taxotron software (Institut Pasteur, Paris, France) to calculate Dice coefficients of correlation and to generate a dendrogram by the unweighted pair group method using arithmetic averages (UPGMA) clustering. The scale indicates the level of pattern similarity. Capital letters indicate macrorestriction types based on visual interpretation of PFGE results. Fragment size (kb) of the reference strain NCTC8325 is indicated on the bottom lane.

### CA-MRSA Antibiotic Susceptibility Profiles

MICs were determined on a selected number of isolates (81) that corresponded to the different PFGE patterns. Overall, CA-MRSA isolates from all locations were susceptible to numerous antimicrobial drugs, including tobramycin, gentamicin, lincomycin, pristinamycin, minocycline, chloramphenicol, ofloxacin, vancomycin, teicoplanin, fosfomycin, rifampin, co-trimoxazole, and linezolid (Table 2). For the purposes of assessing differences between locations, isolates from the United States and Oceania were combined since their susceptibility profiles were similar (Table 2). Minor heterogeneity in erythromycin and mupirocin susceptibility was noted among European isolates. In contrast, U.S. and Oceanian isolates were uniformly susceptible to these antibiotics (Table 2). The main difference between the two groups was their susceptibility to kanamycin, tetracycline, and fusidic acid, with the European group of isolates being more resistant than the U.S. and Oceanian group (Table 2).

**Table 2:** MIC50 and MIC90 of staphylococcal antibiotics against community-acquired methicillin resistant *Staphylococcus aureus* (CA-MRSA) from Europe (46 isolates), United States (22 isolates), and Oceania (13 isolates)

Antibiotics	Isolates from Europe			Isolates from United States and Oceania		
	MIC50 mg/L	MIC90 mg/L	Range mg/L	MIC50 mg/L	MIC90 mg/L	Range mg/L
Benzyl-penicillin	8	8	0.25–8	16	16	4–32
Oxacillin	16	32	4–64	64	64	16–64
Kanamycin	128	128	128	2	2	2
Tobramycin	0.25	0.25	0.25	0.25	0.25	0.25
Gentamicin	1	1	0.5–1	1	1	0.5–2
Erythromycin	0.5	128	0.25–128	0.25	0.5	0.25–128
Lincomycin	0.5	0.5	0.5–32	0.5	0.5	0.25–32
Pristinamycin	0.5	0.5	0.12–1	0.5	0.5	0.12–1
Tetracycline	16	16	0.25–16	0.25	0.25	0.25–32
Minocycline	0.25	0.25	0.25	0.25	0.25	0.25
Chloramphenicol	4	4	4–8	4	8	4–8
Ofloxacin	0.12	0.12	0.12–0.5	0.12	0.25	0.12–1
Fusidic acid	4	4	0.12–64	0.12	0.12	0.12
Vancomycin	0.5	0;5	0.5–1	0.5	0;5	0.5–1
Teicoplanin	0.5	0.5	0.25–0.5	0.25	0.5	0.25–0.5
Fosfomycin	2	2	0.25–2	1	2	0.25–2
Rifampin	0.12	0.12	0.12	0.12	0.12	0.12
Co-trimoxazole	0.5/9.5	0.5/9.5	0.5/9.5	0.5/9.5	0.5/9.5	0.5/9.5
Linezolid	0.5	1	0.25–1	0.5	1	0.25–1
Mupirocin	0.12	0.12	0.12–8	0.12	0.12	0.12

### Comparison of CA-MRSA with HA-MRSA

Using PFGE to compare CA-MRSA isolates with representative HA-MRSA isolates from both France (24 isolates) and the United States (33 isolates), we found that the last isolates grouped into lineages that clearly differed from any of the CA-MRSA isolates of the same continent (not shown). None of the HA-MRSA harbored PVL genes or the SCC*mec* IV element. Moreover, all U.S. HA-MRSA had SCC*mec* type II element; most (31 of 33) of these isolates were *agr* type 2, and the remaining 2 were type 1. Conversely, all French HA-MRSA had an unknown SCC*mec* element according to the method of Oliveira (*15*). These strains were further designated as SCC*mec* IVc by Hiramatsu (*22*). Twenty-three of the 24 French HA-MRSA isolates were *agr* type 1, and only 1 was a type 2. None of the HA-MRSA tested was *agr* type 3, the predominant type for CA-MRSA. In addition, unlike the PVL genes, *lukE-lukD* leukocidin genes were found in most HA-MRSA (95%) as well as in CA-MRSA.

### Analysis of Genetic Background by MLST

Twenty-one representative isolates of each PFGE pattern of CA-MRSA were further characterized by MLST. Overall, the results perfectly matched those of the PFGE with a unique ST corresponding to each group of related PFGE patterns (Table 1). The STs of CA-MRSA were compared with those in the MLST database (Table 3). Within each continent, the most frequent STs of CA-MRSA (i.e., ST1 for the U.S. clone, ST30 for the Southwest Pacific clone, and ST80 for the European clone) were different than the STs of HA-MRSA or methicillin-susceptible *S. aureus* (MSSA) strains in the same continent. For instance, ST1 was detected in U.S. CA-MRSA but only in European MSSA. The only correlation within a continent between the ST of MSSA and CA-MRSA was for the two rare a*gr*1 CA-MRSA clones from the United States (STs 8 and 59). Thus, these two infrequent STs of CA-MRSA were also observed in MSSA (ST8) and HA-MRSA (ST8 and 59) in the same country.

**Table 3:** Origin and frequency of *Staphylococcus aureus* isolates according to their sequence types^a^

Sequence type	Present study		Data from the MLST Web site			
	Country of origin of CA-MRSA	n	Country of origin of MSSA	n	Country of origin of MRSA	n
1	USA	29	UK, Denmark, the Netherlands, Canada,	20		0
8	USA	3	UK, the Netherlands, Denmark, US, Canada	46	Scotland, Ireland, Australia, U.S., UK, Germany, The Netherlands, France	39
30	Oceania (Southwest Pacific clone)	13	UK, Denmark, Germany	83	UK, Spain, Germany, Sweden	8
59	USA	1	UK	4	U.S.	1
80	France, Switzerland	67		0	Greece	1
93	Australia (Queensland clone)	4		0		0

^a^MLST, multi-locus sequence typing ; MRSA, methicillin resistant *S. aureus*; MSSA, methicillin-susceptible *S. aureus*; CA, community acquired.

## Discussion

The characterization of 117 CA-MRSA isolates from three continents indicted four major findings. First, only two genes were unique to CA-MRSA isolates and shared by isolates from all three continents: a type IV SCC*mec* cassette (further designated IVa by Okuma et al. [4]) and the PVL locus. Otherwise, the distribution of the other toxin genes was continent-specific. This finding suggests that PVL and SCC*mec* type IV may confer a selective advantage for community-based MRSA pathogens. Second, CA-MRSA isolates were generally susceptible to most of antibiotics tested apart from β-lactams, although European isolates appeared more resistant (i.e., to kanamycin, tetracycline, and fusidic acid) than U.S. and Oceanian isolates. Third, the genetic background of CA-MRSA organisms was different in each of the three continents, although it was predominantly restricted to the *agr*3 background, which corresponds to one of the three major phylogenetic lineages of pathogenic MSSA previously described (*16*). This finding demonstrates that dissemination of a single CA-MRSA clone did not occur around the world but rather suggests the possibility of simultaneous co-evolution of CA-MRSA organisms in different locations. Fourth, MLST and PFGE analysis showed that within a continent, the genetic background of CA-MRSA strains did not correspond to that of the HA-MRSA in the same continent, suggesting that CA-MRSA did not emerge from local HA-MRSA.

The STs of CA-MRSA clones were not related to the STs of any described pandemic clones of MRSA, such as the Archaic clone (ST250 with a SCC*mec* I element), the Iberian clone (ST247 with a SCC*mec* IA element), the New York/Japan clone (ST5 with a SCC*mec* II element), the Hungarian clone (ST239 with a SCC*mec* III element), the Brazilian clone (ST239 with a SCC*mec* IIIA element), or the pediatric clone (ST5 with a SCC*mec* IV element) (*23*). However, analysis of the MLST database indicated that the CA-MRSA of each continent shared a common genetic background with HA-MRSA or MSSA of other continents (Table 3). This suggests that intercontinental exchange of MRSA or MSSA had occurred, possibly followed by the introduction of the SCC*mec* in MSSA and the PVL locus in MSSA or MRSA. However, we cannot rule out the converse hypothesis that, for instance, MRSA of ST8 from France, which do not harbor the PVL locus and are of *agr*1 allele, derive from an ancestor of *agr*1 allele found in the United States, carrying the PVL locus (Table 3 and data not shown). In any case, the association of SCC*mec* IV (SCC*mec*IVa according to Baba et al. denomination [*6*]) with PVL in the CA-MRSA strains most likely did not result from co-acquisition of the two determinants on a single mobile genetic element because the two loci are widely separated on the *S. aureus* chromosome (*6*).

The CA-MRSA isolates contained the SCC*mec* type IV element, according to the designation of Oliveira and de Lencastre (*15*). If MRSA isolates with the SCC*mec* type IV element contained an additional 381–base pair band because of the integration of pUB110, the isolates are said to be SCC*mec* IVA. However, other groups in Japan and the United States have used region-specific primers to define the L-C region of the SCC*mec* element (*4*). On the basis of L-C polymorphism, researchers have identified three types to date, designated IVa, IVb, and IVc. Thus, the IVa of these latter groups (*4*) is not the same as the IVA of Oliveira and de Lencastre (*15*).

The exact nature of the selective advantage conferred by the observed combination of genetic traits remains to be elucidated, but simple antibiotic selection seems unlikely in a community context of widely different populations with various degrees of methicillin exposure. Okuma et al. (*4*) suggested that CA-MRSA should display enhanced ecologic fitness, as they had a shorter doubling time than HA-MRSA. The real impact of this in vitro observation needs to be evaluated. Unlike other SCC*mec* elements, SCC*mec* IV and SCC*mec* I do not code for additional resistance determinants; however, SCC*mec* IV does code for *mec*A, a peptidoglycan transpeptidase. This protein is expressed at the external surface of the cytoplasmic membrane, where it could interact with the extracellular protein PVL. We are investigating the possibility of PVL activity or of peptidoglycan formation as a result of such an association.

CA-MRSA infections appear to be an emerging phenomenon worldwide. The PVL locus represents a stable genetic marker of these CA-MRSA strains, which explains the frequency of primary skin infections and occasionally necrotizing pneumonia associated with these strains (*2*,*8*,*24*,*25*). Although the selective advantage conferred by the combination of genetic traits (i.e., PVL locus and SSC*mec* IV in an *agr*3 background) is not clear, the spread of a limited number of clones in each continent suggests that these CA-MRSA strains are particularly suited to be successful community-based pathogens.
